# Dopamine D_1_ Agonists: First Potential Treatment for Late-Stage Parkinson’s Disease

**DOI:** 10.3390/biom13050829

**Published:** 2023-05-12

**Authors:** Mechelle M. Lewis, Lauren J. Van Scoy, Sol De Jesus, Jonathan G. Hakun, Paul J. Eslinger, Julio Fernandez-Mendoza, Lan Kong, Yang Yang, Bethany L. Snyder, Natalia Loktionova, Sridhar Duvvuri, David L. Gray, Xuemei Huang, Richard B. Mailman

**Affiliations:** 1 Department of Neurology, Pennsylvania State University College of Medicine, Hershey, PA 17033, USA; 2 Department of Pharmacology, Pennsylvania State University College of Medicine, Hershey, PA 17033, USA; 3 Translational Brain Research Center, Penn State Milton S. Hershey Medical Center, Penn State College of Medicine, Hershey, PA 17033, USA; 4 Department of Medicine, Pennsylvania State University College of Medicine, Hershey, PA 17033, USA; 5 Department of Humanities, Pennsylvania State University College of Medicine, Hershey, PA 17033, USA; 6 Department of Radiology, Pennsylvania State University College of Medicine, Hershey, PA 17033, USA; 7 Department of Public Health Sciences, Pennsylvania State University College of Medicine, Hershey, PA 17033, USA; 8 Department of Psychiatry, Pennsylvania State University College of Medicine, Hershey, PA 17033, USA; 9 Cerevel Neurosciences LLC, Cambridge, MA 02141, USA; 10 Department of Kinesiology, Pennsylvania State University, University Park, PA 16802, USA; 11 Department of Neurosurgery, Pennsylvania State University College of Medicine, Hershey, PA 17033, USA

**Keywords:** dopamine D_1_ agonists, late-stage Parkinson’s disease, dopamine D_1_ receptors, clinical trial

## Abstract

Current pharmacotherapy has limited efficacy and/or intolerable side effects in late-stage Parkinson’s disease (LsPD) patients whose daily life depends primarily on caregivers and palliative care. Clinical metrics inadequately gauge efficacy in LsPD patients. We explored if a D_1/5_ dopamine agonist would have efficacy in LsPD using a double-blind placebo-controlled crossover phase Ia/b study comparing the D_1/5_ agonist PF-06412562 to levodopa/carbidopa in six LsPD patients. Caregiver assessment was the primary efficacy measure because caregivers were with patients throughout the study, and standard clinical metrics inadequately gauge efficacy in LsPD. Assessments included standard quantitative scales of motor function (MDS-UPDRS-III), alertness (Glasgow Coma and Stanford Sleepiness Scales), and cognition (Severe Impairment and Frontal Assessment Batteries) at baseline (Day 1) and thrice daily during drug testing (Days 2–3). Clinicians and caregivers completed the clinical impression of change questionnaires, and caregivers participated in a qualitative exit interview. Blinded triangulation of quantitative and qualitative data was used to integrate findings. Neither traditional scales nor clinician impression of change detected consistent differences between treatments in the five participants who completed the study. Conversely, the overall caregiver data strongly favored PF-06412562 over levodopa in four of five patients. The most meaningful improvements converged on motor, alertness, and functional engagement. These data suggest for the first time that there can be useful pharmacological intervention in LsPD patients using D_1/5_ agonists and also that caregiver perspectives with mixed method analyses may overcome limitations using methods common in early-stage patients. The results encourage future clinical studies and understanding of the most efficacious signaling properties of a D_1_ agonist for this population.

## 1. Introduction

Parkinson’s disease (PD) is characterized clinically by motor and non-motor symptoms. Despite research advances related to disease-modifying therapy, symptomatic treatment using the dopamine precursor levodopa remains the therapeutic cornerstone [1]. Unfortunately, progressive dopamine neuron loss markedly decreases the bioconversion of levodopa to dopamine in the striatum but not mesolimbic areas, thereby decreasing efficacy and increasing side effects [2]. Additionally, potential “off-target” effects from dopamine formation in other monoamine neurons may cause side effects such as drowsiness and hallucinations [3,4,5,6]. A variety of approaches have been used to define these stages of PD, and it is important to note that we follow the nomenclature suggested by Coelho and Ferreira [7], who offered major divisions of early-, advanced-, and late-stage PD. In addition to more disabling motor symptoms (postural instability and falls), late-stage PD (LsPD) patients also experience many non-motor symptoms including anxiety/depression, pain, sleep disorders, cognitive decline, and apathy [8,9], some of which predate motor dysfunction [10].

As PD patients advance to LsPD, there is an increasing family and caregiver burden and higher healthcare costs compared to early- and advanced-stage patients [11,12,13,14,15,16]. There have been no prior controlled drug trials in LsPD patients due partly to the perceived fragility of patients, lack of validated assessments for LsPD, and no accepted target that might mediate symptomatic benefit in patients where levodopa has limited efficacy. As summarized in the Discussion, the post-synaptic cytoarchitecture in LsPD patients is largely preserved despite dopamine neuron degeneration. Thus, targeting post-synaptic dopamine receptor populations could theoretically offer marked therapeutic benefits.

Dopamine receptors were first differentiated into two pharmacological classes, D_1_ and D_2_ [17,18], and cloning a decade later yielded five genes [19]. Of particular importance to this study was the cloning of the D_1_ receptor by the late Professor Mark Caron and others [20,21,22,23]. Many “dopamine agonists” have been approved for human use, and while having some utility in the early stages of PD, they have inferior efficacy to levodopa and intolerable side effects in LsPD [24,25,26]. The term “dopamine agonist”, however, is misleading—currently approved “dopamine agonists” are selective for dopamine D_2_-like receptors (Appendix A.4, Table A1) [25,27,28].

Promisingly, there is compelling neurobiological and pharmacological evidence for the potential of D_1_ receptor-selective agonists to have efficacy in LsPD [24,29,30]. This includes experimental data in severe MPTP-treated non-human primates (NHPs) [31,32] and mid-stage PD patients [33,34]. The early experimental D_1_ agonists, however, contained a catechol-moiety that resulted in significant pharmaceutical liabilities [24]. Newer D_1_ agonists have overcome this limitation [35] and shown efficacy in early- or advanced-stage PD patients [36,37,38,39]. It is unclear, however, if they will have the same low therapeutic index that current “dopamine agonists” have in LsPD patients.

The accessibility to the orally available D_1/5_ partial agonist PF-06412562 (henceforth PF-2562) allowed us first to evaluate the safety of a D_1/5_ agonist in a very short (two-day) feasibility phase I pilot study of LsPD patients [40]. We now explore the efficacy of PF-2562 from that study using several a priori postulates. The first was that the primary dependent variable was caregiver impressions [41,42,43,44] because these individuals were most familiar with patient behavior and daily functioning. Moreover, accepted clinical metrics are not designed to capture meaningful changes in patients with LsPD who had severe multi-domain disabilities and were only permitted to take the drug for two days. Second, we used a convergent mixed methods design involving both quantitative and qualitative data to assist in this goal [45]. We now report the first-ever interventional trial in LsPD that tested the hypothesis that a selective D_1_ agonist, unlike current dopamine agonists or levodopa, may improve the treatment of LsPD.

## 2. Methods

### 2.1. Study Design, Subjects, and Randomization

This study was conducted at PennStateHealth (PSH) in compliance with the Declaration of Helsinki and guidelines for Good Clinical Practice issued by the International Conference on Harmonization. It was reviewed and approved by the US Food and Drug Administration and PSH Institutional Review Board. All participants and caregivers provided signed informed consent. Details of subject recruitment, inclusion and exclusion criteria, baseline medical, protocol information, and safety data were published in a previous report [40]. Briefly, all participants were recruited from our Movement Disorders clinic or a local PD support group and met published diagnostic criteria.

All LsPD subjects had disease duration >15 y and Hoehn and Yahr (HY) stages ≥IV, either “on” or “off” levodopa. Our criteria adapted the terminology of Coelho and Ferreira [7], but differs from others who have used this term less specifically (e.g., disease duration <5 y and HY stages II–III [46]). As a condition of participation, all subjects were informed that regardless of their response to PF-2562, they would not be permitted to continue PF-2562. After informed consent, participants and caregivers were admitted to the Clinical Research Center (CRC) for four days for two consecutive weeks. To maximize comfort, levodopa/carbidopa (parkinsonian symptoms), acetaminophen (pain), ondansetron (nausea), and diphenhydramine (allergies) were given throughout the study when needed.

**Figure A1:**
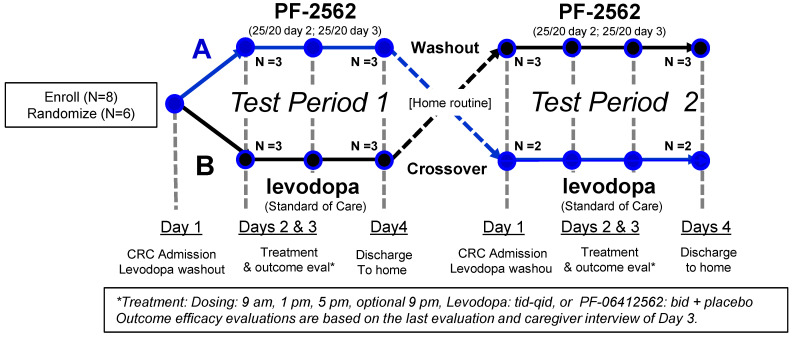
*Study Schematic*.

Eligible participants were randomized to PF-2562 (Sequence A) or levodopa (Sequence B) during Test Period 1 using a 1:1 random allocation sequence and then crossed over to the other drug during Test Period 2 (Appendix A.4, Figure A1). Participants, caregivers, and investigators were blinded to sequence assignment, and participants received identical pill numbers (containing PF-2562, levodopa, and/or placebo) administered at the same time during each sequence. Specifically, following Day 1 baseline evaluation and overnight levodopa/dopamine agonist washout, participants assigned Sequence A received PF-2562 (25 mg at ~0900 h and 20 mg 4 h later) on Days 2–3 during Test Period 1, whereas they received encapsulated Sinemet (carbidopa/levodopa 25/100 mg) 3–4 times (depending on pretrial regimen) 4 h apart on Days 2–3 during Test Period 2. Participants assigned Sequence B received Sinemet in Test Period 1 and PF-2562 in Test Period 2. On Day 4, all participants resumed pre-trial treatment and were discharged after demonstrating no significant complications.

### 2.2. Study Compound Choice

The initial pilot study focused on establishing the safety and tolerability of a D_1/5_ agonist in LsPD, thereby querying the feasibility of conducting clinical trials in LsPD. Among the available D_1/5_ agonists, PF-2562 was selected because it caused acute antiparkinsonian effects in 13 PD patients and was well-tolerated at a 50 mg oral split-dose (t_½_ = 6.4 h, 30 and 20 mg doses four hours apart [37]). This informed the current study design involving a short in-patient stay and cross-over design. Tavapadon, a related D_1/5_ agonist, is titrated to reach efficacious drug levels [39], and the limitations of this pilot study did not allow for extended in-clinic stays to accommodate titration.

### 2.3. Quantitative Data and Metrics

We included five standard quantitative scales [47,48,49,50] for specific efficacy domains representing: motor [MDS-UPDRS motor subscale (MDS-UPDRS-III)]; alertness (Glasgow Coma (GCS) and Stanford Sleepiness (SSS) Scales); and cognition [(Severe Impairment (SIB) and Frontal Assessment (FAB) Batteries]. Scores were obtained three times each on Days 2–3: prior to drug administration and one hour after the first and second doses. We also evaluated sleep using polysomnography (PSG), except in two participants (3 and 4) with deep brain stimulation that disrupts PSG EEG signals. From these data, “sleep efficiency” was selected as the most global/comprehensive metric.

As detailed in our previous report [40], movement disorder clinicians and caregivers completed an adapted validated global clinical impression (GCI) scale designed to assess severity (GCI-S) or change (GCI-C). On Day 1, clinicians evaluated patients’ history and exam (H&P), summarized as a single GCI-S score ranging from 1 = normal/not ill to 7 = extremely ill. Caregivers completed a baseline GCI-S based on their knowledge of the participant’s disease at home that included 17 items summarized as one score ranging from 0–102. At the end of Days 2–3, caregivers and clinicians completed the GCI-C questionnaire that included 17 items scored on a 7-point Likert scale (−3 = marked worsening; 0 = no change; 3 = marked improvement). Clinicians completed this assessment based on interviews with caregivers and daily patient examinations. As pre-specified, Day 3 metrics were used for final analyses to avoid Day 2 confounders such as excitement/noise/environment.

### 2.4. Qualitative Interviews

Qualitative data collection was chosen to capture broad, nuanced experiences, observations, and perspectives of caregivers regarding potential efficacy and/or side effects. Semi-structured caregiver interviews (30–60 min) were conducted by a trained qualitative research assistant at the end of Day 3. Responses were audio-recorded and transcribed verbatim. Interviews explored caregiver-perceived patient response to study drug (if any) and adverse effects compared to patient baseline status. The interview guide used open-ended questions to elicit first general observations from caregivers and then probed specific domains of motor, alertness, cognition, and sleep.

### 2.5. Convergent Mixed Methods Design

Convergent mixed methods designs collect both quantitative and qualitative data for a ‘domain’ and then compare/contrast the conclusions from each dataset (‘merging’) to reach a comprehensive conclusion [51]. At study conception, pre-selected domains were guided by our clinical experiences with LsPD patients and extant literature. Table 1 lists these domains (motor, alertness, cognition, sleep, and clinician/caregiver impression of change) and the quantitative and qualitative measures corresponding to each. Domains were analyzed separately, and conclusions were drawn independently. Blinded data were then integrated by merging findings and seeking points of convergence/divergence in the conclusions. This mixed methods approach establishes stronger credibility and validity to the findings when convergence of conclusions is established and opportunities to extract lessons when divergence is detected [51,52].

### 2.6. Analysis

Quantitative analysis: Quantitative scales provided one score (GCS, SSS, and sleep efficiency) or several that were summed (SIB, FAB, and MDS-UPDRS-III) for each participant. The score on Day 2 prior to the study drug administration was subtracted from the score at the end of Day 3 to evaluate change. Both clinician and caregiver GCI-C scores also were captured. Scores are presented for each participant in this pilot study (detailed descriptions in Appendix A). Based on the pre-determined efficacy assessment, the primary endpoint was caregiver ratings that were analyzed using a paired Student’s *t*-test (two-tailed α = 0.05).

Qualitative analysis: Conventional content analysis, including data transformation, was used to evaluate the data [51]. Published guidelines for methodological rigor of qualitative analysis were followed to ensure attention to the truth-value, applicability, consistency, and neutrality of findings [51,53]. Three independent, blinded analysts used qualitative software (NVivo Ver. 11.0, QSR International, Melbourne, Australia) to code and analyze the data (details in Appendix A).

Mixed methods integration: Joint displays were constructed to compare quantitative efficacy outcomes with results from the transformed qualitative data for each participant completing the study. The study team reviewed conclusions from both the quantitative and qualitative datasets to ascertain an integrated conclusion regarding the preliminary efficacy of PF-2562 [52].

## 3. Results

### 3.1. Participants

Six subjects met the inclusion criteria (demographics in Table 2). Patients had a mean age of 73.5 (±4.5 SD) y, and two participants were female. Consistent with protocol inclusion criteria, patient HY stages all were >4 in the ‘on’ state. No subject required levodopa rescue during the PF-2562 week, whereas one participant received rescue medication during the levodopa week (subject 4, 0.5 100/25 mg levodopa/carbidopa tablet Day 2, 1 Day 3).

Of the six patients who were randomized, one (subject 6, disease duration 19 y) withdrew after the first arm because of blood pressure fluctuations the clinical team felt were related to the interaction of the test drug with baseline dehydration, related kidney dysfunction, and autonomic dysfunction [40]. This patient’s data are excluded from these efficacy analyses. The remaining five patients completed both arms of the study.

Key narrative phrases from caregiver interviews qualitatively described the patient’s baseline functional status (Table 2). Four of five patients (subjects 1, 3, 4, and 7) represented classic LsPD patients and had disease durations of 15–23 y. All patients had been treated with symptomatic drugs and two with deep brain stimulation. In addition to motor disability and requirement of walker and/or wheelchair use, all patients had varying challenges maintaining normal sleep/wake cycles and being alert/engaged during the daytime, and all displayed clinically significant cognitive dysfunction.

Subject 8 had the longest disease duration (32 y). All drugs, including levodopa, had caused intolerable side effects, and thus, this patient had not been treated with any Parkinsonian drugs for three years prior to study enrollment. On most days, he was in unarousable “deep sleep”, but able to reflexively suck/swallow if his mouth was stimulated with a straw or food in a more “awake” state. Because of his atypical background and long survival without dopaminergic medication, we highlight his response to treatment in subsequent sections since it may provide unique insight into LsPD pathophysiology.

### 3.2. Quantitative Results

**Figure 1:**
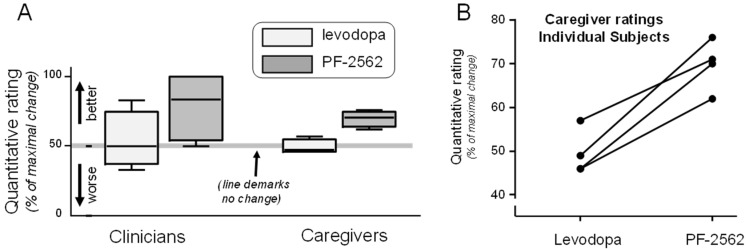
**Evaluation of quantitative GCI data for four of five subjects:** (**A**) Clinician (left) and caregiver (right) quantitative global clinical impression (GCI) of change scores on Day 3 of the levodopa (light gray) or PF-2562 (dark gray) week. The horizontal line indicates no change, with scores above the line reflecting better scores. Clinician scores were more variable than those from caregivers for both levodopa and PF-2562, but both favored PF-2562. (**B**) Quantitative GCI scores from the caregivers representing the four classic LsPD patients rated PF-2562 consistently better than levodopa (*p* = 0.007).

Standardized scales assessing motor function, alertness, cognition, and sleep did not detect a clear pattern of differences between levodopa and PF-2562 (Table 3 and Appendix A). Clinician scores were more variable than those from caregivers for both levodopa and PF-2562 (Figure 1A), and caregivers rated PF-2562 consistently better than levodopa (*p* = 0.007; Figure 1B). This offered initial evidence that PF-2562 may provide improved efficacy based on caregiver scores [40]. As expected from LsPD patients and the short duration of D_1/5_ agonist treatment, there was no significant improvement in severely affected motor function as assessed by MDS-UPDRS-III (Appendix A.4, Table A2).

### 3.3. Qualitative Caregiver Interview

Blinded analysis of the transcripts revealed significant variability in patients’ baseline functional status (Table 4). Notably, caregivers did not distinguish explicitly among alertness, attention, and cognition according to qualitative analyses. Therefore, these domains collapsed as ‘patient overall engagement’ in the mixed methods joint display. Results of the qualitative data transformations (improved, worsened, unchanged) are shown in Table 4, along with quotations from caregivers describing the changes they noticed within each domain. Overall, the qualitative data suggested PF-2562 improved cognitive engagement and motor domain status (balance, weakness, and rigidity) in the four typical LsPD subjects.

Qualitative analyses also suggested PF-2562 may improve facial expression and sleep to varying degrees, although analysis of sleep was challenging due to highly variable caregiver descriptions (e.g., the judgment of sleep quality based on different aspects such as breathing, apneas, duration, depth of napping, restlessness, vocalizations). All caregivers commented that some environmental factors may have impacted results. For example, caregiver-1 said: “*I would attribute some of the alertness… to the rigid schedule [that] does keep him at his best…the constant stimulation of people is different than at home*”. Similarly, caregiver-7 noted: “*Here the chair styles are a little bit different, a little deeper and the floors are a little slicker, footwear was a little different*”.

Subject 8 responded dramatically to levodopa but not PF-2562 (see Table 3 and Table 4). Prior to unblinding, both the clinician and caregiver felt Test Period 2 (levodopa) was far superior to Test Period 1 (PF-2562). After a discussion with the research ethics consult service, we decided it was our responsibility to convey these results to the family. This was performed, and the patient’s family decided to restart levodopa. They reported levodopa had no beneficial effect, and the patient remained in a “deep sleep” state.

### 3.4. Mixed Methods Results

Integration of quantitative and qualitative data suggested a convergent finding that caregivers favored PF-2562 in four of five patients who completed the study (Table 5). Caregiver observations suggested alertness and engagement/cognition domains had the most dramatic changes in the four typical LsPD participants. Caregivers also noted that environmental factors likely influenced the improvements during both weeks. Additionally, the qualitative data uncovered a potential side effect not measured discretely in questionnaires (‘twitching’) or detected on quantitative measures. This observation was reported during both the levodopa and PF-2562 testing periods. No caregivers or patients commented specifically on dyskinesia or a special “feeling” that would suggest they were taking levodopa.

Caregivers were consistent in their quantitative observations, whereas clinician impressions displayed substantial variability and diverged from caregiver impressions in two of five patients. The rater-dependent standard metrics detected no differences and were not contributory to the overall results.

## 4. Discussion

LsPD patients have many unmet needs, and supportive and palliative care has increasingly been recognized as the best options, e.g., reviews [54,55]. This first controlled interventional study in LsPD patients explored the potential benefits of a D_1/5_ agonist exceeding palliative care in this population [32]. We included caregiver perspectives and used mixed methods [56] to identify efficacy domains of PF-2562 based on the premise that: (1) traditional clinical tools would be relatively insensitive given the small sample size and short duration/evaluation period and (2) PD patients and their neurologists differ markedly in assessing physical, psychological, and other domains that predict the quality of life (QoL) [7,57]. Our data showed caregivers captured potential benefits of PF-2562 in LsPD patients in ways standard clinical metrics did not. Moreover, mixed methods allowed the transformation of semi-structured caregiver observations to quantifiable metrics and identified key domains of improvement (motor, alertness, and cognitive engagement) that warrant future attention. We provide additional information about the caregiver perspective and mixed methods used in Appendix A. Together, the results encourage more future clinical studies, as well as an understanding of the most efficacious signaling properties of a D_1_ agonist for this population.

### 4.1. Mechanisms of Action of Levodopa vs. D_1_ Agonist in LsPD

**Figure 2:**
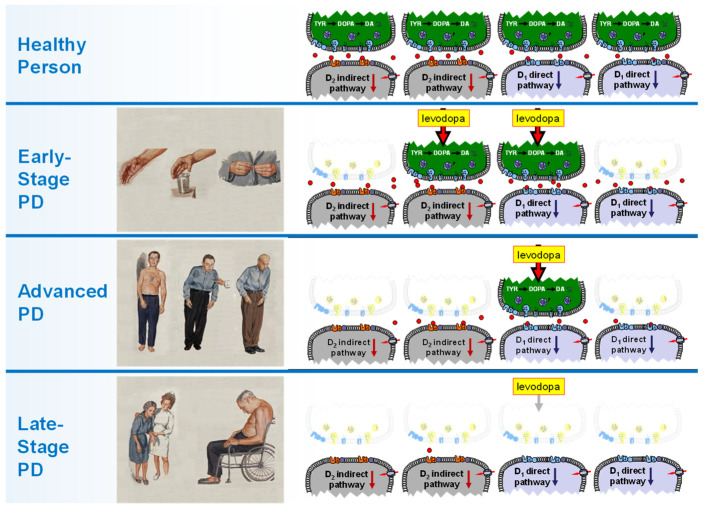
**Synaptic mechanisms explaining the loss of levodopa effectiveness with PD progression.** Levodopa is an indirect dopamine agonist that must be converted to dopamine in residual nerve terminals. It is estimated that 40–60% of the terminals are lost at first diagnosis (Early-stage PD). By late-stage illness, >>90% of terminals have degenerated, preventing production of dopamine in critical areas of the basal ganglia (also see Figure 3).

As a prodrug, levodopa must first be converted to dopamine. Data from animal models and in vivo and post-mortem data from PD patients show that levodopa treatment causes a dramatic increase in total dopamine in the BG [58,59,60]. Because dopamine has metabotropic actions (i.e., functions as a “slow” neuromodulator) at both synaptic and extrasynaptic/volume receptors, levodopa is a very effective therapy because the remaining terminals work “overtime” to process levodopa. Consistent with this concept, dopamine terminal density decreases far more than bulk terminal metabolism [61]. *This is manifested clinically as the “honeymoon” period in early-stage PD (EsPD), where levodopa causes a balanced activation of post-synaptic dopamine receptors (Figure 2).*

As PD progresses, there is continued nigral neuronal death and fewer presynaptic nigrostriatal terminals that can process levodopa [62]. **As the patients enter advanced PD (AdvPD, Figure 2),** the remaining terminals that had worked “overtime” in EsPD no longer can supply dopamine adequately. Raising the dose of levodopa fails to improve basal ganglia function because of the lack of terminals, whereas the increased levodopa dose activates extra-basal ganglia dopamine receptors in the mesolimbic system causing psychiatric and other side effects.

Despite continuous nigrostriatal degeneration, the mesolimbic and mesocortical dopamine systems are less damaged during PD progression [2], and the administration of levodopa results in higher than normal concentrations of dopamine in terminal regions like the nucleus accumbens [2,63]. *The resulting mesolimbic hyperdopaminergic state is what we believe leads to many of the problems (e.g., hallucinations, sleepiness) seen in LsPD patients treated with levodopa [64,65].* **From this conceptualization, basic pharmacological principles predict that a dopamine agonist should be able to restore normal function, assuming that other aspects of the motor system are still functional.**

**Figure 3:**
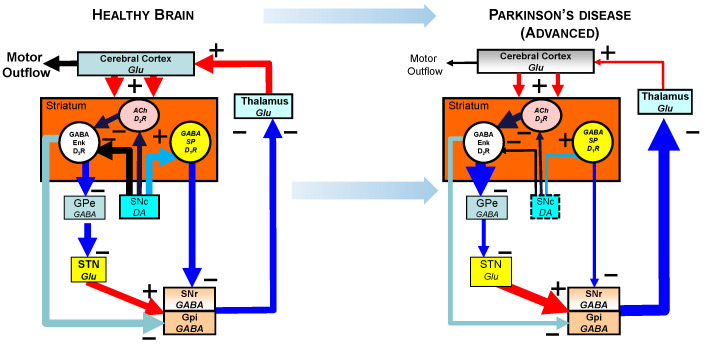
**Schematic of basal ganglia chemoarchitecture in healthy and PD brains.** Levodopa, an indirect dopamine agonist, must be converted to dopamine in nerve terminals. In normal brain, the circuitry of the basal ganglia balances activation of the direct and indirect pathways to stimulate the motor cortex (**left** panel). In EsPD, 40–60% of terminals are lost. By LsPD, >90% of terminals have degenerated, preventing production of dopamine in critical areas of basal ganglia. The result is a dramatic increase in inhibitory signaling to the thalamus that then decreases the stimulation of the motor cortex needed to initiate and maintain motor function (**right** panel).

Receptor localization and systems circuitry relevant to LsPD. The rationale for testing D_1/5_ agonists is based on classical studies of basal ganglia circuitry [24,66,67,68,69,70,71]. Specifically, D_1_ receptors are highly segregated and expressed at high concentrations on medium-spiny GABA neurons of the direct pathway (Figure 3). Our hypothesis has been that the input of the indirect pathway becomes the primary regulator of outflow to the thalamus, with the continued loss of dopaminergic innervation that is >90% in LsPD.

### 4.2. Unresolved Mechanisms in These Findings

As noted earlier, in NHP models [31] and PD itself [33], selective full D_1_-like agonists are at least equally effective as levodopa. Importantly, the full D_1/5_ agonist dihydrexidine markedly attenuated parkinsonian motor signs in MPTP-treated NHPs with disability analogous to subjects in the current study, whereas neither levodopa nor the D_2/3_ agonist bromocriptine was effective [32]. In NHP models equivalent to AdvPD, the partial D_1/5_ agonist PF-06649751 (now CVL-751) was equieffective to levodopa and with lower dyskinesia liability [72]. We know of no study in very disabled NHPs that compared a partial with a full agonist.

The compound we used, PF-2562, is one of a series of non-catechol D_1/5_ agonists originally discovered by Pfizer. Two others (tavapadon and CVL-751) are in Phase II and III clinical trials and are reported to have ca. 70% and 55% D_1_ canonical (i.e., cAMP) intrinsic activity, with PF-2562 being less efficacious. Interestingly, as a series, these new drug candidates were reported to have no intrinsic activity at D_1_-mediated β-arrestin recruitment [36,73]. We have confirmed this for PF-2562 by comparing it to dopamine and dihydrexidine, the first full CNS-available D_1_ agonist [74].

Intriguingly, dihydrexidine, like its 2-methyl analog [75], are “super-agonists” at D_1_-mediated β-arrestin recruitment (Table 6). Whether the latter property is an artifact of these assay systems or physiologically meaningful is yet to be determined, but the lack of D_1_ β-arrestin in vitro recruitment activity is consistent with the previous literature [36,73]. Assuming that the data we have produced in NHP and now in LsPD are replicable by others, several key pharmacological questions will be important to answer.

The two most obvious are what are the optimal ligand properties in terms of intrinsic activity at canonical and non-canonical pathways to provide the highest therapeutic index. For example, it has been argued that the lack of β-arrestin activity will decrease desensitization due to chronic administration, yet it is also possible the receptor occupation needed to obtain antiparkinson effects will be too low in vivo to trigger these mechanisms. In that case, the non-canonical signaling of β-arrestin (or other unstudied pathways) may be very important.

The availability of newer generation D_1/5_ agonists renewed a broad interest in targeting D_1_-like receptors to improve cognitive function in multiple disease states [76]. The current data provide tantalizing evidence that this benefit may extend to LsPD patients. As with motor signs, increased apathy as PD progresses also is observed commonly [77]. PD apathy and impulse control disorders may be opposite motivational expressions caused by hypo- and hyperdopaminergia, with apathy resulting from hypodopaminergia along with anhedonia, anxiety, and depression. Since the approved D_2_/D_3_ agonists are relatively ineffective, the current data suggest D_1_ agonists also may be effective for motivational deficits in LsPD.

## 5. Conclusions and Future Directions

The current authors [78,79,80], like the late Professor Caron [81], have had a keen awareness of how the signaling properties of a drug, as well as the drug’s affinity profile, could markedly affect physiological effects in vitro and in vivo. The D_1/5_ agonists currently in phase III trials differ in these properties from the experimental D_1/5_ agonists that had been widely used over decades in the laboratory and in a few limited clinical trials. These pharmacological differences (both intrinsic activity at canonical pathways and functional selectivity) [73,82] will be important to investigate in future studies involving both clinical populations and NHP models of severe Parkinsonism.

As a first-of-its-kind, the current study is limited by its relatively small sample size. As experience with D_1/5_ agonists in LsPD is gained, there may be ways to select compounds with specific profiles to gain maximal therapeutic benefit [80,83,84]. In addition, we have used the term D_1/5_ agonist throughout this paper since there are no small biomolecules that are adequately selective for either of these two subtypes. In the primate striatum, the D_1_ receptor is expressed at very high levels and almost exclusively on GABAergic medium spiny neurons of the direct pathway. Conversely, D_5_ expression is very sparse and appears only on cholinergic interneurons of the indirect pathway. We believe the antiparkinson effects are due to those direct pathway D_1_ receptors, but additional studies are needed to verify this hypothesis.

It is also important to address the one subject who had a profound positive response to levodopa. Subject 8 previously had been essentially unresponsive to all treatment for years, suggesting it was not a random event. The dramatic improvement during the levodopa week might represent a re-sensitization to levodopa after a three-year “drug holiday”, but this seems unlikely since there was no effect when the family resumed levodopa. Although highly speculative, another hypothesis is that the two-day PF-2562 period “primed” dopamine circuitry (e.g., by improving sleep structure) to respond more normally to even small amounts of dopamine from a levodopa challenge six days later. Coupled with the very consistent beneficial responses of the other four patients, the hypothesis is a high priority for further testing, as there will be a growing number of LsPD patients with better palliative care strategies, which may increase the life-span, but not the health span of PD patients.

We have noted the limitations of this study above, but it is important to put them in context. There was no prior experience in the literature for interventional studies in LsPD. Thus, necessarily, the design [40] focused on safety and feasibility, limiting both subject numbers and permitted treatment duration (i.e., two days). Efficacy was a second primary endpoint that could be evaluated (as we now report) only if the a priori safety concerns allowed the study to go to completion. Despite these limitations, the study was extremely rigorous: it was completely blinded to all except the research pharmacists; it was placebo-controlled for all medications; it used a cross-over comparison design: the analysis plan was decided a priori, and all data were locked and blinded until opened by the statistician. The finding of a significant improvement required excluding the data from subject 8. We feel this was justified, as described earlier. It is noteworthy that for the other four subjects, PF-2562 could have worsened them or had no effect, yet all had meaningful improvement when compared to standard-of-care levodopa. We feel these data provide compelling evidence for further investigation into the potential value of D_1/5_ agonists in LsPD using increased numbers of subjects and longer drug administration periods. Such studies must incorporate caregiver perspectives that should be conducted at home to eliminate environmental influences on patient behavior. If these findings are confirmed, they will dramatically affect the lives of PD patients at a stage of disease for which there has been little hope and no prior experimentation.

## Figures and Tables

**Table 1:** Convergent mixed methods study design: Constructs and Measures.

Construct	Quantitative Measure	Qualitative Measure (Caregiver Interviews)
Motor	MDS-UPDRS-III-motor subscale	Tell me about [patient]’s normal level of *[alertness, cognition, facial expression, movement or rigidity, sleep]*. How has *[patient]’s [alertness, cognition, facial expression, movement or rigidity, sleep]* been over the past two days? Tell me about that. **PROMPTS (if needed)**: Can you give some examples of things that you have noticed? How is [patient]’s level of *[alertness, cognition, facial expression, movement or rigidity, sleep]* different compared to a week ago? How, if at all, has this changed since the infusion started? When did you notice these changes? Have you noticed these kinds of changes before? Tell me more about that.
Alertness	Glasgow Coma scale (GCS) Stanford Sleepiness Scale (SSS)	
Cognition	Severe Impairment Battery (SIB) Frontal Assessment Battery (FAB)	
Sleep	Sleep efficiency	
Overall	Clinician Global Clinical Impression of Change (GCI-C) modified for late-stage stage PD patients Caregiver Global Clinical Impression of Change (GCI-C) questionnaire	How do you think [patient] responded to the treatment over the past two days? Can you give some examples of things that you have noticed? How, if at all, has [patient] changed since the infusion started?

[] includes domain-specific words. Abbreviations: MDS-UPDRS-III-Movement Disorders Society Unified Parkinson’s Disease Rating Scale motor exam.

**Table 2:** Demographic, clinical history, and baseline data for the randomized participants.

ID	Demographic, Key Medical and Surgery Milestones	Current Medication	Disease Stages and Severity *	Caregiver Perspective Key Narrative/Phrases from Qualitative Interviews
1	M, PD at 36–40 y; pallidotomy at 46–50 y; wheelchair use at 61–65 y; PEG at 61–65 y	DA drugs: Parcopa; Non-DA drugs: rivastigmine transdermal	HY stage: 5; MD-GCI-S: 6 CG-GCI-S: 96	Periods of intermittent wakefulness between mid-am to later-pm, less alert after levodopa. Requires two people to help him out of bed, stiff in am. Incontinent at baseline.
3	M, PD at 55–60 y; STN-DBS at 66–70 y; walker and wheelchair use at 76–80 y	DA drugs: Sinemet R and CR; Non-DA drugs: Vitamin B12	HY Stage: 4–5; MD-GCI-S: 4 CG-GCI-S: 37	Trouble with concentration, often not remembering things. Discomfort with social interactions, doesn’t smile, appears sad. Sleepy after drugs. Hard time getting up, freezing. Uses a chair lift. Often days & nights ‘mixed up’.
4	F, PD at 56–60 y; STN-DBS at 61–65 y; Walker use at 66–70 y; Wheelchair use at 66–70 y	DA drugs: Sinemet R & CR, Rytary, selegiline, pramipexole; Non-DA Drugs: dexlansoprazole, melatonin, midodrine, donepezil, memantine, clozapine, rimantadine, methylphenidate, venlafaxine fludrocortisdone	HY Stage: 4–5; MD-GCI-S: 5 CG-GCI-S: 52	Confusion, peaks and valleys, emotional, a blank facial expression. Often urgency and incontinence and constipation. Sleeps deeply >14 h/day. Has a lot of dreaming and vocalization, particularly in second half of the night. Nocturnal movements wax and wane. Some difficulty with swallowing pills.
6	F, PD at 56–60 y; Walker use at 76–80 y; Wheelchair at 76–80	DA drugs: Rytary, Sinemet; Non-DA drugs: gabapentin, donepezil, lorazepam, quetiapine, melatonin, tramadol	HY Stage: 4–5; MD-GCI-S: 4 CG-GCI-S: 10	Some mild short-term memory problems. Naps 3–4 h per day, frequent awakenings at night with vocalizations. Mild swallowing problems if she eats too quickly.
7	M, PD at 51–55 y; Cane use at 71–75 y; Wheelchair use at 71–75 y	DA drugs: Rytary, rasagiline; Non-DA drugs: donepezil	HY Stage: 4–5; MD-GCI-S: 4 CG-GCI-S: 38	Varying in alertness, doesn’t communicate much with facial expressions, shows strong emotions occasionally. Stooped posture with head tilted right. Issues with frozen foot. Yells in sleep, frequent dreams.
8	M, PD dx at 41–45 y; Levodopa was stopped due to severe drossiness at 66–70 y; Bed-bound at 66–70 y	DA drugs: none; Non-DA drugs: none	HY Stage: 5: MD-GCI-S: 6 CG-GCI-S: 64	Sleeps for days at a time, not very cognitive when awake, has difficulty verbalizing. Does not connect with others or TV or music. Does not move, feed, or hold anything. Lacks facial expression. Vocalizes in dreams, occasionally move leg in sleep.

All subjects aged ≥66 y at time of enrollment. Abbreviations: CGI-S: Clinical global impression of disease severity rated by a movement disorder (MD) specialist or caregiver (CG); DA: Dopaminergic; Dx: Diagnosis; F: Female; HY: Hoehn and Yahr; M: Male; PEG: Percutaneous endoscopic gastrostomy; Sinemet: carbidopa/levodopa; R: regular release; CR: controlled release. * MD-GCI-S was rated by clinician at baseline on Day 1, from 1 to 7, 1 = normal to 7 = extremely ill. CG-GCI-S = 17-item scale rated by caregiver on Day 1 with each item rated from 1 = normal to 7 = extremely ill. Maximal score was 102. Parcopa, Sinemet, and Rytary are proprietary formulations of levodopa. These data were originally reported in [40].

**Table 3:** Summary of quantitative data.

**Motor Function**								
	**UPDRS-III ** **(+Score Better)**		**Clinician**		**Caregiver**			
	**Levodopa**	**PF-2562**	**Levodopa**	**PF-2562**	**Levodopa**	**PF-2562**		
**1**	−2	16	2	1	0.2	0.6		
**3**	4	3	1	3	−0.3	1.7		
**4**	1	−2	0	2	−0.2	1		
**7**	−1	−2	0	0	−0.4	0.4		
**8**	−14	−24	2	4	2.2	0.4		
**Alertness**								
	**GCS (+score better)**		**SSS (−score better)**		**Clinician**		**Caregiver**	
**ID**	**Levodopa**	**PF-2562**	**Levodopa**	**PF-2562**	**Levodopa**	**PF-2562**	**Levodopa**	**PF-2562**
**1**	2	−3	−1	1	2	2	2	3
**3**	0	0	0	0	0	2	0	3
**4**	1	−1	−1	1	1	3	−1	2
**7**	0	0	−1	0	0	0	0	1
**8**	−6	−3	−4	−2	2	0	3	1
**Cognitive function**								
	**SIB (+score better)**		**FAB (+score better)**		**Clinician**		**Caregiver**	
**ID**	**Levodopa**	**PF-2562**	**Levodopa**	**PF-2562**	**Levodopa**	**PF-2562**	**Levodopa**	**PF-2562**
**1**	0	0	−3	0	1	1	0	0.7
**3**	−1	1	1	−3	0	1	0	1.7
**4**	−1	1	3	−3	−1	3	−0.5	1.5
**7**	0	0	1	−2	0	0	−0.3	0
**8**	0	0	−3	0	1	0	1.7	0.2
**Sleep**								
	**SE (+score better)**		**Clinician**		**Caregiver**			
	**Levodopa**	**PF-2562**	**Levodopa**	**PF-2562**	**Levodopa**	**PF-2562**		
**1**	−3.4	−9.4	2	0	1	3		
**3**	N/A	N/A	0	0	0	2		
**4**	N/A	N/A	0	0	0	0		
**7**	0.5	−6	0	0	−1	2		
**8**	33.5	32.7	2	0	1	0		

Abbreviations: FAB: Frontal assessment battery; GCS = Glasgow coma scale; SE: sleep efficiency; SSS = Stanford sleepiness scale; SIB: Severe impairment battery; MDS-UPDRS-III: Movement Disorders Society Unified Parkinson’s Disease Rating Scale, subscore III. Quantitative data in each domain first represent standard clinical instruments for measuring that domain. The scores represent the difference between the last measure on Day 3 (2 h after second dose of study medication) and the first measure on Day 2 (prior to administration of any study medication). For the GCS (best score = 15), SIB (best score = 133), and FAB (best score = 18), higher scores represent better performance, whereas, for the SSS (best score = 1) and MDS-UPDRS-III (best score = 0), higher scores represent worse performance. The global clinical impression of change (GCI-C) in each domain was assessed at the end of Day 3 by the movement disorder specialist (clinician) or caregiver. The caregiver score is the average of several checklist items related to that domain: Alertness: 1 item; Cognition: 4 items; Motor: 5 items; Sleep: 1 item. The following scale was used: +3-Marked improvement, +2-Moderate improvement, +1-Minimal improvement, 0-No change, −1-Minimal worsening, 2-Moderate worsening, −3-Marked worsening. Shading for the standard clinical instruments indicates whether the scores improved (light gray) or worsened (dark gray) for levodopa and PF-2562. For the clinical and caregiver GCI-C scores, the shading indicates which treatment was favored (light gray favored, dark gray not favored). No shading represents no change in scores (standard clinical instruments) or equivocal scores (GCI-C ratings).

**Table 4:** Qualitative data transformation and quotations (PF-2562 in grey cells).

		Data Transformation	Additional Qualitative Insights	Quotes
**Subject 1**	**Levodopa**	Improved cognitive engagement (alertness/cognition) Improved motor and strength Either mildly improved or no ∆ swallowing	CG notes that patient was more alert and social than at home, but also attributes this to a change in environment and rigidity of schedule, increased stimulation from staff. Patient looking around room, calling staff by name, and had improved facial expressions and movements. More closed mouth (‘peaceful’) breathing. Unclear if changes in sleep or napping.	*“…he is certainly more alert and aware, however it’s comparable to when he has a really good day at home” * *“…the high point was…, where he picked the hat up and… trying very hard to put it on…he reached out and took hold of [research assistant’s] hand,…looked at him,….attended to him, and… asked [him] for the hat…that was probably the most…purposeful activity we have seen in a while”.*
	**PF-2562**	Improved cognitive engagement (alertness/cognition) Improved motor and strength Either mildly improved or unchanged swallowing	Has had some moments of alertness at home, but not nearly as long as here. Try to speak, had improved alertness and cognition, better movements and strength when pushing things away. Jerking movements of arms. Less drooling. Unclear if changes in sleep or napping	*“…I was enjoying the alertness and interaction during, and it was so long…really good to have him that alert” “… he definitely engaged [more}…if you spoke to him, he would turn back… those moments are shorter at home”.* *“He could set his foot up so his knee was up high,… cross his legs, and I have seen him at home struggle to cross his legs… there are a couple of very purposeful things that actually worked both yesterday…& this afternoon”.*
**Subject 3**	**Levodopa**	No ∆ cognitive engagement (alertness/cognition), movement Wax & wane in facial expression No ∆ swallowing or breathing	Overall, no major changes Needs assistance with balance, standing, walking Worse toe tapping	*“I would say [alertness has been] the same as at home”. * *“We went around last evening and he froze up a good bit…the same as at home…instead of [MD] just holding on to him a little…, he did okay, but it’s not like last [PF] week… just the same as home, he’s not real steady. Somebody definitely has to hold on to him or he’d fall”.*
	**PF-2562**	Improved cognitive engagement (alertness/cognition) Improved facial expression Improved movement and muscle weakness No ∆ swallowing or breathing	Much improved walking compared to home, able to do side steps, he was ‘walking right along’ (with MD) Improved mood and alertness; able to pay attention and follow along with a TV show Smiling for first time in 2 years Less messy eating, eating well with a spoon	*“I see his personality today. like before he got Parkinson’s…he was just a lot of fun and [came] up with wise cracks and stuff and he was just like his old self today…”* *“We couldn’t believe how good he was walking here. Even made the side steps to come back and get on his chair again, so that was definitely an improvement from home”.*
**Subject 4**	**Levodopa**	No ∆ in facial expression Mild/slight improved cognitive engagement (alertness/cognition), movement	Difficult to assess changes because she commonly has peaks and valleys Wax and wane at baseline. Somewhat improved focus on walking	*“…it’s not terribly far off from home…I would say that on average she has been as good if not just a hair better here”.* *“I would say slightly better here…but even here, she is off crashing into things”.*
	**PF-2562**	No ∆ in cognitive engagement (alertness/cognition) or waxing and waning Worse facial expression	Appears less erratic, less waxing and waning. More consistent focus, less distraction on her tasks Very deep sleep, nearly unresponsive, urinated in bed	*“She is more consistently off– there are still ups and downs but it’s less distant between the peaks and valleys”.*
**Subject 7**	**Levodopa**	Worse cognitive engagement (alertness/cognition) Worse movements No ∆ in balance No ∆ in bladder	Overall, more lethargic and worse cognition, although some improved alertness on day 2 that CG attributes to posture in chair and new setting; became more lethargic once acclimated, hard to arouse Twitching and jumpy during sleep	*“We could not arouse him….he was a little bit interactive with the ice water and then [research assistant] finally just got real in his face and started talking to him in that man voice… that was the first time he opened his eyes. It was taking him longer sometimes to come up with what he wanted to say”.* *“He was twitching and jumping…I have never seen that”.*
	**PF-2562**	Improved cognitive engagement:(alertness/cognition) and facial expression. Worse movements and strength Improved balance	Better mood and interaction, felt ‘energized’ and ‘optimistic’ although notes some grogginess on Day 2. Interactions and stimulation have been helpful. Slower movements and muscle weakness More frequent urination, sensed need to go	*“He said ‘I feel energized, I feel excited about today. I feel like doing things’ a couple of times”.* *I think the movements are a little bit slower than when he is on his typical [meds]…the pace has been pretty slow but…he hasn’t been losing his balance. He has been…much better today” [with regards to balance].*
**Subject 8**	**Levodopa**	Improved cognitive engagement (alertness/cognition) Improved facial expressions Improved movements Unclear ∆ twitching	Dramatic response in alertness, responsiveness, memory, and communication Shook someone’s hand to greet them, able to move more Became more tired and lethargic as day wore on	*“Today it seemed to change completely. He made conversation, he greeted people…he responded to questions and could bring up some memories and verbalize them… it’s a big change today”.* *“That’s something a little new [twitching], it’s not that he has never done it at home… he jerks, but, yeah, he has been twitching and jerking quite a bit here…I wouldn’t say it’s increased necessarily. Today right now we are seeing quite a bit of it, but last week was more”.*
	**PF-2562**	No ∆ or improved cognitive engagement (alertness/cognition) No ∆ facial expression No ∆ swallowing Waxing and waning movements and rigidity	Worse twitching of arms and legs No major changes noted by CG CG notes increased stimulation from environment	*“[He] has been very stimulated… so many people coming and going and all the activity, a lot more than he gets at home…but I don’t think it is any different than what he would have responded to before”.* *“I don’t see a big change in [movement or muscle tone]……he’s been very stiff, very rigid, um, but I think this morning he was a little looser…When he was examined, things seem to be better, but he’s back to being very stiff and rigid”. “Um…he was very twitchy today which was something new”.*

Abbreviations: CG: Caregiver.

**Table 5:** Mixed methods integrated joint display merging quantitative and qualitative data and conclusions.

Domain of Interest	Quantitative			Qualitative	Data Integration	Conclusion
	Rater	Clinician	Caregiver			
	Scale	GCI	GCI	Interview		
Motor	Equivocal	Equivocal	Favored PF-2562 in first 4 subjects	Favored PF-2562 in first 4 subjects	PF-2562 was superior to levodopa, according to caregiver data.	Standard and clinician-based evaluations are equivocal. Caregiver data converge in 4/5 patients, favoring PF-2562. Key efficacy domains are motor, alertness, and engagement/cognition. Last subject has unique features and responses, which shall analyze and discuss separately.
Alertness	Equivocal	Equivocal	Favored PF-2562 in first 4 subjects	Favored PF-2562 in first 4 subjects	PF-2562 was superior to levodopa, according to caregiver data.	
Cognition	Equivocal	Equivocal	Favored PF-2562 in first 4 subjects	Favored PF-2562 in first 4 subjects	PF-2562 was superior to levodopa, according to caregiver data.	
Sleep	Incomplete	Equivocal	Equivocal	Equivocal	Sleep data is incomplete and equivocal between the two drugs.	

**Table 6:** Intrinsic activity of PF-2562 and reference ligands.

Ligand	E_max_ Adenylate Cyclase Stimulation (% Dopamine)	E_max_ β-Arrestin Recruitment (% Dopamine)
PF-2562	41 ± 15%	ND
Dopamine	100 ± 9%	100 ± 3%
Dihydrexidine	103 ± 14	210 ± 25

Studies performed in D_1_-transfected CHO cells using the GloSensor cAMP assay. β-arrestin recruitment assay performed using the DiscoverX Pathfinder kit.

**Figure A2:**
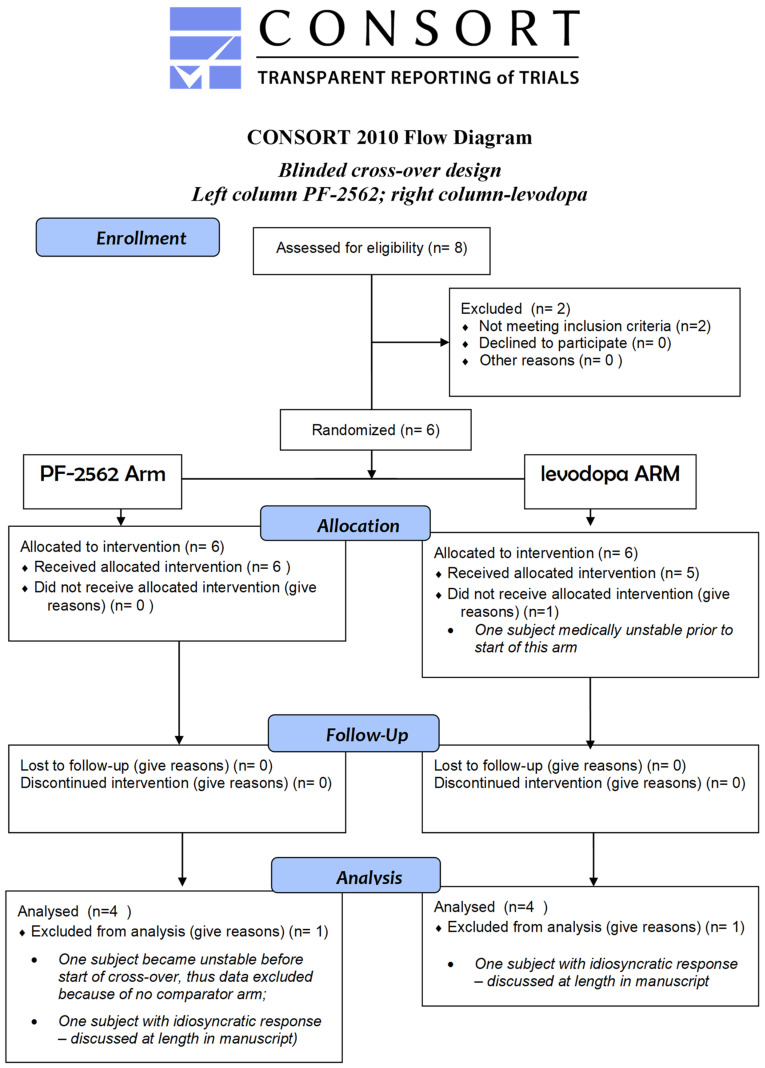
*Consort Flow Diagram*.

## Data Availability

Additional details are available in supplemental data at https://www.medrxiv.org/content/10.1101/2022.04.30.22270885v1. The data are not publicly available due to privacy/ethical restrictions, but de-identified data are available upon reasonable request to the corresponding author X.H. All requests must be in writing, and the identity of the requestor will be confirmed.

## Appendix A

### Appendix A.1. Details of Quantitative Analysis

**Standard scales:** As noted in the text, the standardized, validated scales assessing alertness, cognition, motor function, and sleep did not detect a significant pattern of differences between levodopa and PF-2562. The clinician ratings also were equivocal (Table 3). **For alertness,** levodopa improved GCS scores in two participants (1 and 4), worsened them in one (8), and had no effect in two (3 and 7), whereas PF-2562 worsened scores in three participants (1, 4, and 8) and had no effect in two (3 and 7). Levodopa improved SSS scores in 4 participants (1, 4, 7, and 8) and had no effect in one (3), whereas PF-2562 improved SSS scores in one participant (8), worsened two (1 and 4), and had no effect in two (3 and 7). **For cognition,** levodopa worsened SIB scores for two participants (3 and 4) and had no effect in three (1, 7, and 8), whereas PF-2562 improved SIB scores in two participants (3 and 4) and had no effect in three (1, 7 and 8). Levodopa improved FAB scores in three participants (3, 4, and 7) and worsened them in two (1 and 8), whereas PF-2562 worsened FAB scores in three participants (3, 4, and 7) and had no effect in two (1 and 8). **For motor function,** levodopa improved MDS-UPDRS-III scores in two participants (3 and 4) and worsened them in three (1, 7, and 8), whereas PF-2562 improved MDS-UPDRS-III scores in two participants (1 and 3) and worsened them in three (4, 7, and 8). **For sleep efficiency,** levodopa improved scores in two participants (7 and 8) and worsened them in one (1), whereas PF-2562 improved sleep efficiency in one participant (7) and worsened it in two (1 and 8).

**Clinician CGI Ratings**: For alertness, clinician GCI-C scores favored levodopa in one participant (8), PF-2562 in two (3 and 4), and were equivocal in two (1 and 7; Table 3). Clinician GCI-C scores for cognition favored levodopa in one participant (8), PF-2562 in two (3 and 4), and were equivocal in two (1 and 7). For motor function, GCI-C scores favored levodopa in one participant (1), PF-2562 in three (3, 4, and 8), and were equivocal in one (7). Clinician GCI-C scores for sleep efficiency favored levodopa in two participants (1 and 8) and were equivocal in three (3, 4, and 7).

**Caregiver CGI Ratings:** For alertness, caregiver GCI-C scores favored levodopa in one participant (8) and PF-2562 in four (1, 3, 4, and 7; Table 3). Caregiver GCI-C scores for cognition favored levodopa in one participant (8) and PF-2562 in four (1, 3, 4, and 7). For motor function, caregiver GCI-C scores favored levodopa in two participants (1 and 8) and PF-2562 in three (3, 4, and 7). Caregiver GCI-C scores for sleep efficiency favored levodopa in one participant (8) and PF-2562 in four (1, 3, 4, and 7).

### Appendix A.2. Details of Qualitative Analysis

As noted in the text, a conventional content analysis approach that included data transformation was used to evaluate the data [51]. Published guidelines for methodological rigor of qualitative analysis were followed to ensure attention to the truth-value, applicability, consistency, and neutrality of the findings [51,52]. Three independent, blinded analysts used qualitative software (NVivo Ver. 11.0, QSR International, Melbourne, Australia) to code and analyze the data. **First,** a preliminary codebook was developed inductively based on the common concepts that emerged from the data. The codebook followed closely the structured interview domains yet also included unexpected categories and concepts that were included in the final codebook. **Second,** the preliminary codebook was applied to an additional three transcripts, and minor codebook adjustments were made to fit the additional data. Some domains were collapsed as appropriate based on the data. Data saturation (the point at which no new codes emerge) was achieved after reviewing 6 of 12 transcripts (one per participant per treatment week), and the final codebook contained codes for each of the key efficacy domains as well as caregiver observations from the home and study environments. **Third,** the finalized codebook was then used to recode the entire dataset by two coders. Codes were adjudicated by a third analyst to ensure inter-rater reliability. Discrepancies were reconciled via group discussions. **Finally,** analysts used data transformation to convert the qualitative data into categories (i.e., improved, worsened, and remained unchanged) for each domain based on the codebook. Any differences in coding were reconciled by group discussion [85].

### Appendix A.3. Importance of Caregiver Perspectives

Most clinical trials rely upon informed clinician judgment based on validated instruments and (when available) imaging/molecular/biochemical markers, but no validated standard scales exist for LsPD [7]. Clinical ratings of complex behaviors necessarily involve short evaluation epochs with inherent inter-individual and inter-location variability. Prizer et al. [57] found PD patients, and their neurologists differed markedly in assessing physical, psychological, and other domains predicting QoL, and the value of caregiver input has been recognized previously [41,42,43,44,57,86]. In our study, alertness, social interaction, and QoL improvements reported by caregivers reflect changes that are critical to palliative care in LsPD. Decreasing apathy, increasing arousal, or similar improvement in non-motor and motor function could have wide applicability in the absence of “cures”.

Caregiver observations also were more consistent and less variable than experienced physicians. This is not surprising since caregivers were intimately familiar with nuanced baseline patient behaviors, were able to provide insight and context for typical/atypical behavioral observations, and were with participants 24/7 during the study. It also is noteworthy that blinded caregivers consistently identified the levodopa week as not being remarkably different from home. This gives credence to the caregivers’ observations and objectivity.

### Appendix A.4. Mixed Methods in a Phase I Study

Mixed method approaches in clinical trials often are limited to pre-trial use or assessing implementation issues such as recruitment [87] and seldom have been used to examine drug trial outcomes [88]. Our approach revealed efficacy endpoints and observations not captured by questionnaires with pre-specified areas of inquiry or anticipated prior to study initiation. The qualitative data added texture to quantitative caregiver evaluations, and their convergence provides compelling data for additional studies investigating PF-2562 to enhance both motor function and cognitive engagement. Future studies should consider integrating mixed method strategies at the phase I stage that may lead both to cost savings and a more effective selection of efficacy endpoints in phase II–III trials.

Schematic of the overall design of the study (modified from [40]). Subjects were randomized to receive PF-2562 followed by levodopa (Sequence A, top) or levodopa followed by PF-2562 (Sequence B). PF-2562 (5 mg) or placebo tablets were provided by Pfizer. Sinemet (carbidopa/levodopa, 25/100 mg) tablets were encapsulated to preserve the study blind. The bottom part of the schematic shows the events that occurred on each day. Levodopa dose was based on home dosage and regimen. Some subjects received a fourth dose of levodopa if that was required according to their pre-trial dosing regimen. Outcome efficacy data are based on the last evaluation and caregiver interview of Day 3.

**Table A1:** *Direct or Indirect Dopamine Receptor Agonists That Have Been Approved or Are in PD Clinical Trials*.

*Drug Class*	*Target(s)*	*Current Status*	*Clinical Effects*	*Side Effects*
**Levodopa** ***(indirect dop-*** ***amine agonist)*** [levodopa/carbidopa-based combinations or formulations]	Results in dopamine that targets all dopamine receptors At higher doses, may affect “off-target” receptors due to “off-site” DA	Standard-of-care for Parkinson’s disease since 1967 *(formulations include Sinemet, Parcopa, Duopa, Rytary; Stalevo; Bendopa, Inbrija)*	Very effective in early and mid-stage disease	More side effects with disease progression (dyskinesias, on-off; hypotension; drowsiness and hallucinations in later stages)
**“D_1_ agonists”** **(D_1_–D_5_ non-selective)**	Dihydrexidine (full agonist)	Non-human primates; Phase Ib	Very effective in severely PD non-human primates	Severe hypotension in humans; short-acting; injectable only
	ABT-431 (full agonist)	two published Phase II trials	Very effective in mid-stage PD	Hypotension; nausea; injectable only
	PF-06412562 (PF-2562)	Phase IIa	Effective in mid-stage PD	Hypotension; nausea
	tavapadon (PF-06649751)	Phase III	Effective in mid-stage PD	Hypotension; nausea
**“Dopamine agonists”** ***(selective D_2_/D_3_)***	cabergoline (Dostinex)	(Withdrawn, valvulopathy)	Moderate efficacy (does not match levodopa); used for earlier stage PD and as adjuvant	Hypotension; obsessive and compulsive disorders; Drowsiness; hallucinations
	pramipexole (Mirapex)	Approved drug		
	ropinirole (Requip)	Approved drug		
**“Dopamine agonists”** ***(selective D_2_/D_3_ with some D_1_ affinity)***	bromocriptine (Parlodel)	Approved drug; *D_1_ antagonist*	Moderate efficacy	Hypotension; obsessive and compulsive disorders; Drowsiness, hallucinations
	pergolide (Permax)	Withdrawn (valvulopathy); *D_1_ partial agonist*	More effective than bromocriptine	
	rotigotine (Neupro)	Approved drug (patch)	Patch only	
	apomorphine (Apokyn)	Approved drug (injection or sublingual)	Short-acting; effective for rescue	

**Table A2:** *MDS-UPDSR-III Scores*.

		Mean ± SD
**Levodopa**	Baseline	83.7 ± 26.2
	Day 2, time 2	81.6 ± 24.7
	Day 2, time 3	84.8 ± 22.8
	Day 3, time 2	88.2 ± 27.2
	Day 3, time 3	82.2 ± 25.6
**PF-2562**	Baseline	81.5 ± 19.5
	Day 2, time 2	90.0 ± 26.1
	Day 2, time 3	83.8 ± 27.4
	Day 3, time 2	79.4 ± 30.1
	Day 3, time 3	82.8 ± 28.7

Average Movement Disorder Society Unified PD Rating Scale (MDS-UPDRS) III (motor) subscores for each treatment condition (levodopa or PD-2562) at each timpoint of the study. Neither levodopa nor PF-2562 had a significant impact on MDS-UPDRS-III motor scores (traditional scale) at any timepoint.

CONSORT flow diagram of the study design. A total of eight participants were enrolled in the study (Enrollment) and six were randomized. Five participants completed both study arms, with one withdrawn after the first week due to them being medically unstable (Allocation and Follow-Up). Data from four traditional late-stage PD participants were analyzed, with one subject having an idiosyncratic response that is discussed at length in the manuscript.
